# Survival benefits of gastrectomy in gastric cancer patients with stage IV: a population-based study

**DOI:** 10.18632/oncotarget.22535

**Published:** 2017-11-20

**Authors:** Xingkang He, Sanchuan Lai, Tingting Su, Yangyang Liu, Yue Ding, Sheng Quan, Jianmin Si, Leimin Sun

**Affiliations:** ^1^ Department of Gastroenterology, Sir Run Run Shaw Hospital, Zhejiang University Medical School, Hangzhou, China; ^2^ Institute of Gastroenterology, Zhejiang University (IGZJU), Hangzhou, China; ^3^ Department of Gastroenterology, The Second Affiliated Hospital of Zhejiang University Medical College, Hangzhou, China; ^4^ Department of Clinical Medicine, Zhejiang University City College, Hangzhou, China

**Keywords:** metastatic gastric cancer, gastrectomy, SEER, survival analysis

## Abstract

**Objective:**

The aim of the current study is to investigate the role of gastrectomy for survival among metastatic gastric cancer patients.

**Results:**

We finally identified 12,986 eligible patients with stage IV GC between 2004 and 2012, including 1,981 (15.3%) patients with gastrectomy and 11,005 (84.7%) without surgery. The median overall survival time for patients with and without surgery were 9.0 (95%, 8.3–9.7) and 4.0 (95%, 3.9–4.1) months respectively. Patients who received gastrectomy had a significantly better survival outcome compared with those without surgery (*P* < 0.05). In the multivariate Cox analysis, gastrectomy was associated with decreased overall mortality (HR, 0.47, 95% CI 0.44–0.49, *P* < 0.001) and cancer-specific mortality (HR, 0.46, 95% CI 0.44–0.50, *P* < 0.001). The survival benefits associated with surgery persisted even after performing the propensity score matching analysis (overall survival, HR, 0.47, 95% CI 0.43–0.50, cancer-specific survival, HR, 0.47, 95% CI 0.44–0.50).

**Conclusions:**

Based on population-based study, we demonstrated that there was a survival advantage of gastrectomy in stage IV GC patients. Further prospective trials need to verify our findings.

**Materials and Methods:**

We included an eligible cohort of stage IV gastric cancer (GC) patients in the Surveillance, Epidemiology and End Results (SEER) database from 2004 to 2012. The survival difference of patients with and without gastrectomy were assessed by Kaplan–Meier analysis and log-rank test. Multivariate Cox analyses were performed to analyze the effect of gastrectomy on overall and cancer-specific mortality. Furthermore, we performed propensity score matching (PSM) to reduce the potential selection bias.

## INTRODUCTION

Gastric cancer remains a leading cause of cancer-related death globally [1, 2]. According to GLOBOCAN 2012 estimated, there were 951,000 new diagnosed cases, which accounts for 723,000 cancer-related deaths globally in 2012 [2]. With widespread eradication of *H. Pylori* and early detection, the morbidity and mortality of gastric cancer had been declining rapidly in the past few decades [3–7]. However, the prognosis of patients with metastatic diseases was still dismal and most cases survived less than one year [8]. Compared with favourable prognosis of localized stage (nearly 95% 5-year survival), the 5-year survival rate (SR) for advanced cancer varied from 10% to 20% [9, 10].

In the United States, most of gastric cancer patients were diagnosed at stage IV and died from the distant metastasis ultimately [11–13]. Current therapies for metastatic gastric cancer mainly included chemotherapy, consisting of fluoropyrimidine/ cisplatin-based combination regimens [14]. Although radical gastrectomy was the first choice for early gastric cancer [15], the value of gastrectomy in stage IV remains a great controversy. Several studies had demonstrated that gastrectomy could prolong the survival of patients, improve quality of life and benefit from alleviation of cancer-related complications [16–18]. Conversely, other studies indicated that an unfavourable overall survival after resection in patients with metastatic gastric cancer [19–22]. They stated that gastrectomy was associated with higher complication rates and mortality rates, which even impeded systemic chemotherapy and decreased quality of life [23–25]. Limited number of samples in previous studies might limit their generalization. For incurable gastric cancer, palliative resection is only recommended for patients presented with cancer-related complications (such as bleeding, obstruction or perforation) [26]. At present, there was limited data from prospective or randomized clinical trials to address the potential impact of gastrectomy on survival of patients with metastatic diseases.

Therefore, we aimed to conduct this population-based study to explore whether gastrectomy of the primary tumour leads to overall and cancer-specific survival benefits, which might expand on current existing knowledge.

## RESULTS

### Patient baseline characteristics

**Figure 1:**
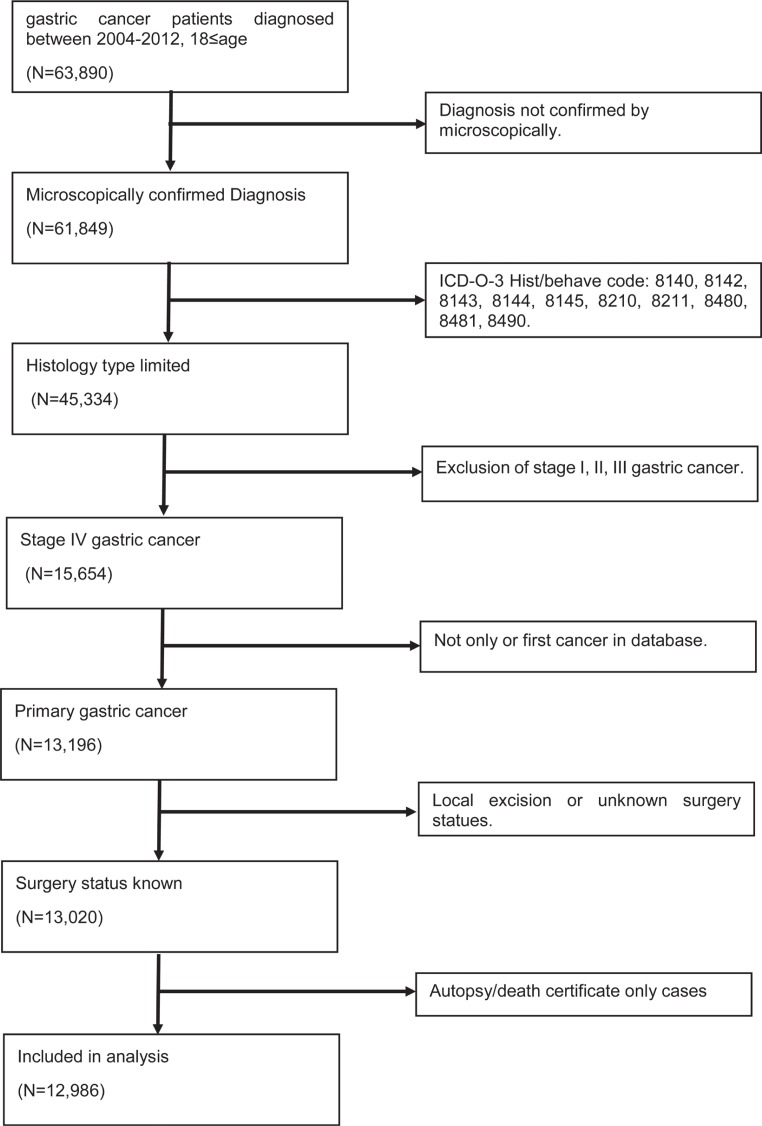
Selection of gastric cancer patients with stage IV in the study

**Figure 2:**
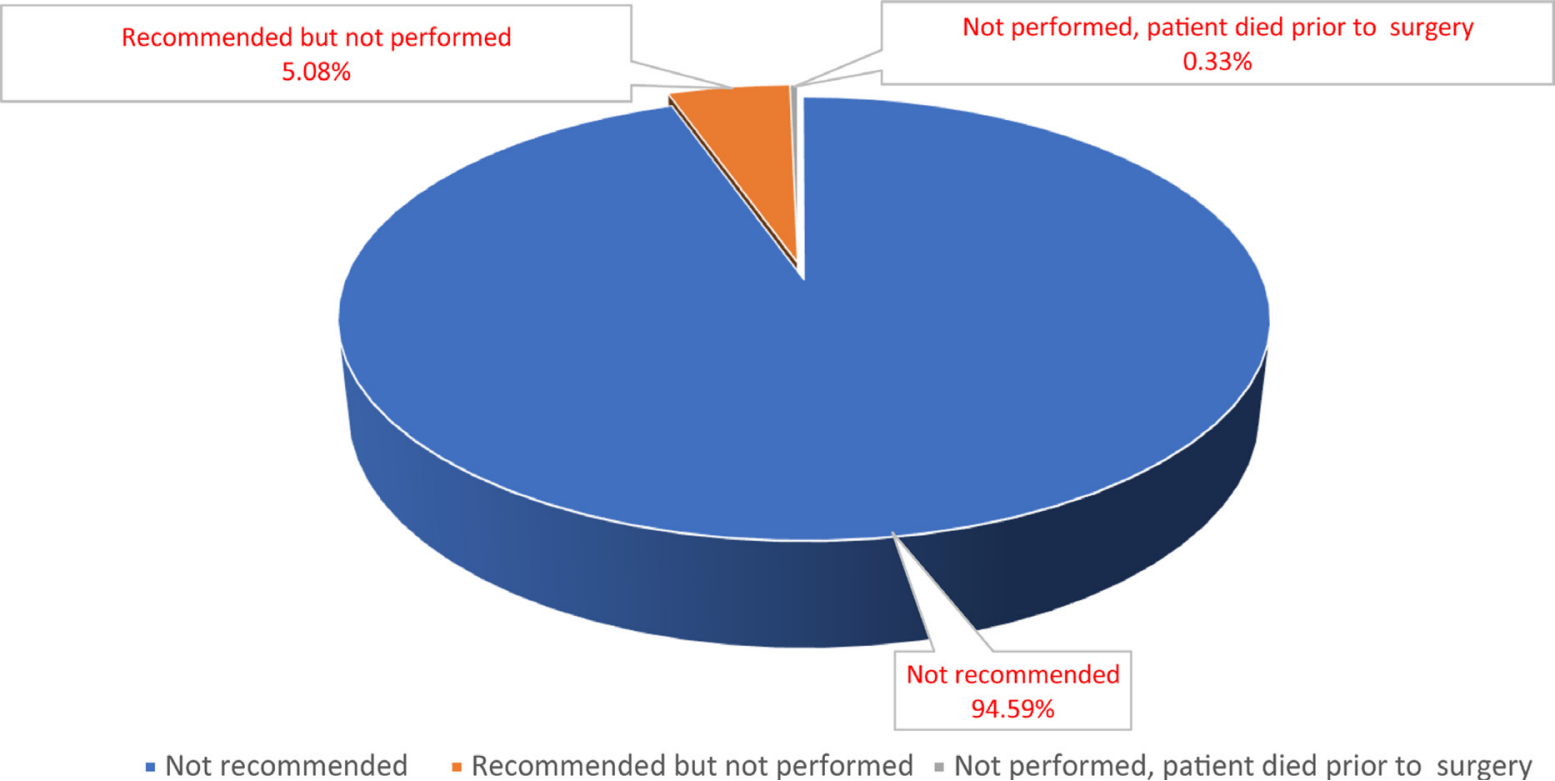
Distribution of reason that patients did not undergo surgery

**Figure 3:**
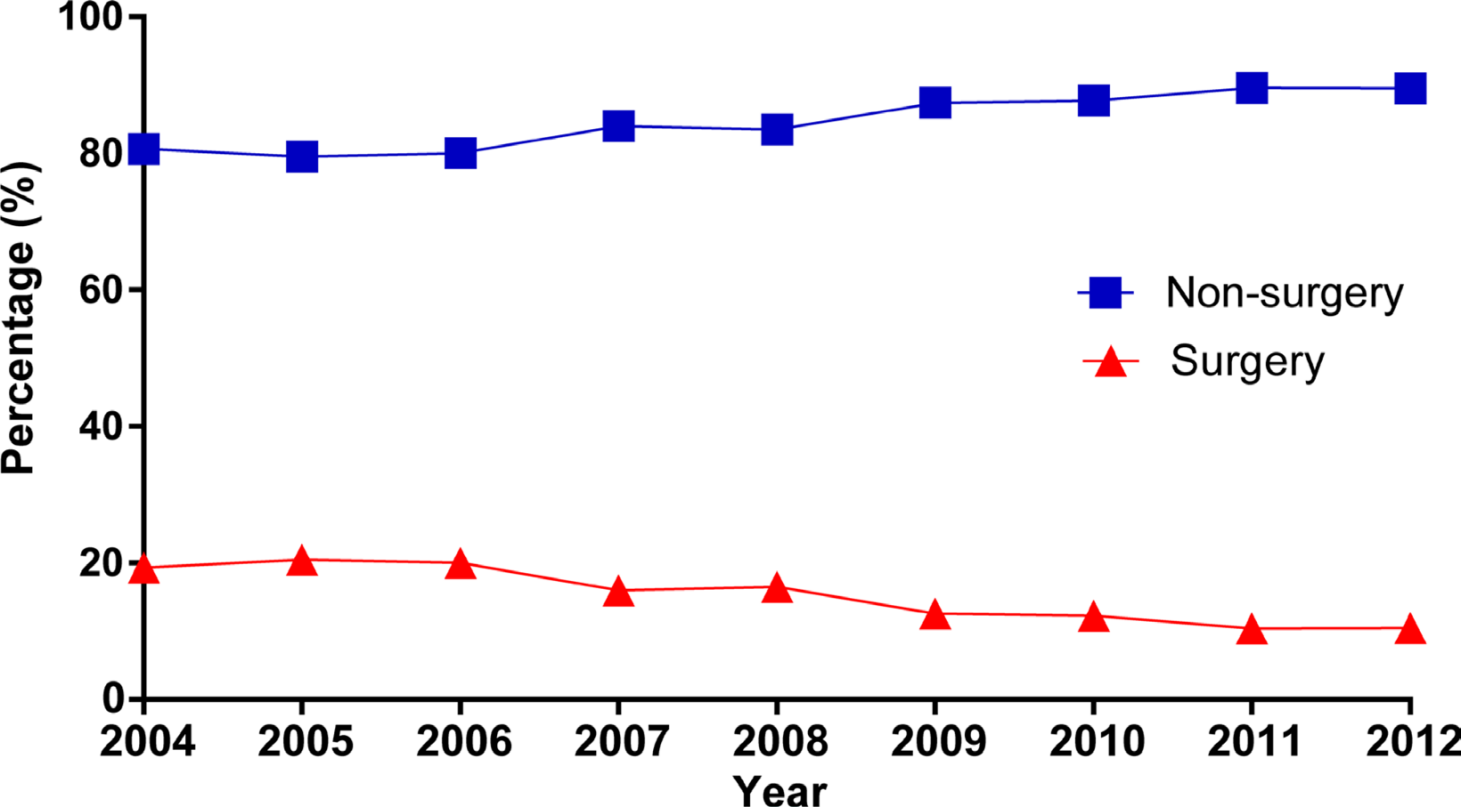
The trend of treatment options in stage IV gastric cancer from 2004 to 2012

According to our inclusion criterion, totally 12,986 eligible patients, including 1981 (15.3%) patients underwent gastrectomy (surgery group) and 11005 (84.7%) patients did not undergo any surgery (non-surgery group) (Table 1). The detailed selection procedure of eligible patients was summarized in Figure 1. For patients who underwent surgery, total (or near-total) gastrectomy was carried out in 30.8% (*N* = 610) of total population and non-total gastrectomy was performed in remaining patients. For patients without gastrectomy, we analyse the underlying reason about no cancer-directed surgery. As Figure 2. shown, most patients were not recommended to received surgery and 5.17% of population was recommended to undergo gastrectomy, yet did not have surgery for other reasons. From 2004 to 2012, we observed a decreasing trend of gastrectomy rate in patients with stage IV GC (From 19.3% to 10.5%, Figure 3). Compared with the non-surgery group, those who had undergone surgery were more likely to be younger and married, have tumour with poor differentiation.

**Table 1:** Patient baseline characteristics of gastric cancer patients with stage IV

Characteristics	Total raw data (*N* = 12,986)	Patient characteristics in raw data (*N* = 12,986)			Patient characteristics after PSM (*N* = 3962)		
		No Surgery (*N* = 11,005)	Surgery (*N* = 1,981)	*P*	No Surgery (*N* = 1981)	Surgery (*N* = 1981)	*P*
**Year of diagnosis**				<0.001			<0.001
2004–2006	4,192 (32.3%)	3,357 (30.5%)	835 (42.2%)		667 (33.7%)	835 (42.2%)	
2007–2009	4,291 (33.0%)	3,646 (33.1%)	645 (32.6%)		661 (33.4%)	645 (32.6%)	
2010–2012	4,503 (34.7%)	4,002 (36.4%)	501 (25.2%)		653 (33.0%)	501 (25.3%)	
**Gender**				<0.001			0.347
Male	8,191 (63.1%)	7,019 (63.8%)	1,172 (59.2%)		1,201 (60.6%)	1,172 (59.2%)	
Female	4,795 (36.9%)	3,986 (36.2%)	809 (40.8%)		780 (39.4%)	809 (40.8%)	
**Age**				0.003			0.747
<60	5,004 (38.5%)	4,181 (38.0%)	823 (41.5%)		813 (41.0%)	823 (41.5%)	
≥60	7,982 (61.5%)	6,824 (62.0%)	1,158 (58.5%)		1,168 (59.0%)	1,158 (58.5%)	
**Insurance Status**				<0.001			<0.001
Uninsured	552 (4.3%)	495 (4.5%)	57 (2.9%)		95 (4.8%)	57 (2.9%)	
Insured	8,049 (62.0%)	6,989 (63.5%)	1,060 (53.5%)		1,198 (60.5%)	1,060 (53.5%)	
Unknown	4,385 (33.8%)	3,521 (32.0%)	864 (43.6%)		688 (34.7%)	864 (43.6%)	
**Race**				<0.001			0.027
White	9,431 (72.6%)	8,100 (73.6%)	1,331 (67.2%)		1,398 (70.6%)	1,331 (67.2%)	
Black	1,706 (13.1%)	1,423 (12.9%)	283 (14.3%)		231 (11.7%)	283 (14.3%)	
Other	1,849 (14.3%)	1,482 (13.5%)	367 (18.5%)		352 (17.8%)	367 (18.5%)	
**Marital status**				<0.001			0.033
Unmarried	5,064 (39.0%)	4,372 (39.7%)	692 (34.9%)		684 (34.5%)	692 (34.9%)	
Married	7,431 (57.2%)	6,204 (56.4%)	1,227 (61.9%)		1,260 (63.6%	1,227 (61.9%)	
Unknown	491 (3.8%)	429 (3.9%)	62 (3.1%)		37 (1.9%)	62 (3.1%)	
**Grade**				<0.001			0.361
Well/Moderate differentiated	2,518 (19.4%)	2.146 (19.5%)	372 (18.8%)		351 (17.7%)	372 (18.8%)	
Poor differentiation/ Undifferentiated	7,798 (60.1%)	6,309 (57.3%)	1,489 (75.2%)		1,525 (77.0%)	1,489 (75.2%)	
Unknown	2,670 (20.6%)	2,550 (23.2%)	120 (6.1%)		105 (5.3%)	120 (6.1%)	
**Tumour site**				<0.001			0.883
Cardia	4,364 (33.6%)	4,019 (36.5%)	345 (17.4%)		360 (18.2%)	345 (17.4%)	
Body	1,204 (9.3%)	1,026 (9.3%)	178 (9.0%)		181 (9.1%)	178 (9.0%)	
Lower	3,578 (27.6%)	2,634 (23.9%)	944 (47.7%)		916 (46.2%)	944 (47.7%)	
Overlapping lesion of stomach	1,285 (9.9%)	1,031 (9.4%)	254 (12.8%)		268 (13.5%)	254 (12.8%)	
Stomach NOS	2,555 (19.7%)	2,295 (20.9%)	260 (13.1%)		256 (12.9%)	260 (13.1%)	
**Chemotherapy**				0.270			0.355
No	5,982 (46.1%)	5,092 (46.3%)	890 (44.9%)		919 (46.4%)	890 (44.9%)	
Yes	7,004 (53.9%)	5,913 (53.7%)	1,091 (55.1%)		1,062 (53.6%)	1,091 (55.1%)	
**Radiation therapy**				0.127			0.581
No	10,914 (84.0%)	9,272 (84.3%)	1,642 (83.0%)		1,655 (83.5%)	1,642 (82.9%)	
Radiation	2,072 (16.0%)	1,733 (15.8%)	339 (17.1%)		326 (16.5%)	339 (17.1%)	

Abbreviation, PSM Propensity Score Weighting, NOS, Not Otherwise Specified.

### Surgery as a prognostic factor for survival

**Figure 4:**
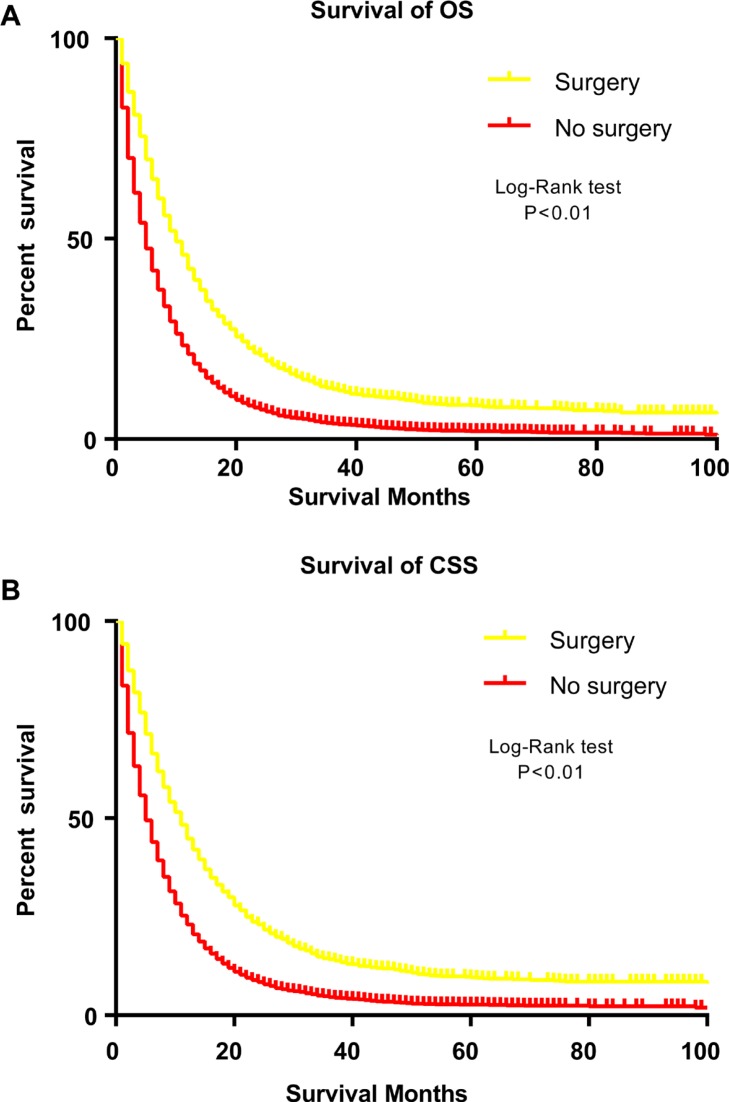
Survival analysis of patients with stage IV gastric cancer by Kaplan-Meier curves before propensity matching procedure (**A**) overall survival. (**B**) cancer-specific survival.

The one-year overall SR was 43.5% for the surgery group and 20.1% for the non-surgery group. For cancer-specific survival, the one-year SR for patients with or without gastrectomy was 46.3% and 22.8%, respectively. The Kaplan–Meier curves further illustrated that there were significant differences regarding OS and CSS between patients with or without surgery (Figure 4A, 4B). Subsequently, crude Cox analysis revealed that surgery indicated better OS (HR, 0.55, 95% CI 0.52, 0.58) and CSS (HR, 0.54, 95% CI 0.52, 0.57). After adjustment of other variables, gastrectomy remained a significant prognostic factor for overall (HR, 0.47, 95% CI 0.44–0.49) and cancer-specific survival (HR, 0.46, 95% CI 0.44–0.50; Table 2). In addition, other variables, such as age, race, marital status, tumour grade, site, chemotherapy and radiation were also considered as prognostic factors. Stratified analyses were performed to demonstrate the prognostic impact of gastrectomy by age, chemotherapy and radiation (Table 3). The survival benefits of gastrectomy were not influenced by these variables. Furthermore, we also excluded patients who were not recommended to surgery in non-surgery group to reduce selection bias. In multivariable Cox analysis, gastrectomy still significantly decreased risk for all mortality (HR, 0.56, 95% CI, 0.51–0.62) and cancer-specific mortality (HR, 0.55, 95% CI, 0.49–0.61)

**Table 2:** Prognostic factors for overall and cancer-specific mortality in patients with advanced disease^a^

Variable	Overall Survival				Cancer-specific Survival			
	Crude		Multivariate		Crude		Multivariate	
	HR (95% CI)	*P*	HR (95% CI)	*P*	HR (95% CI)	*P*	HR (95% CI)	*P*
**Year of diagnosis**								
2004–2006	Reference		Reference		Reference		Reference	
2007–2009	0.91 (0.87, 0.95)	<0.001	NA	NA	0.90 (0.86, 0.94)	<0.001	NA	NA
2009–2012	0.89 (0.85, 0.93)	<0.001	NA	NA	0.88 (0.84, 0.92)	<0.001	NA	NA
**Gender**								
Male	Reference		Reference		Reference		Reference	
Female	1.04 (1.00, 1.08)	0.041	0.95 (0.92, 0.99)	0.012	1.05 (1.01, 1.09)	0.022	NA	NA
**Age**								
<60	Reference		Reference		Reference		Reference	
≥60	1.28 (1.24, 1.33)	<0.001	1.11 (1.07, 1.16)	<0.001	1.26 (1.21, 1.31)	<0.001	1.10 (1.05, 1.14)	<0.001
**Insurance Status**								
Insured	Reference		Reference		Reference		Reference	
Uninsured	NA	NA	NA	NA	NA	NA	NA	NA
**Race**								
White	Reference		Reference		Reference		Reference	
Black	1.09 (1.03, 1.15)	0.002	1.08 (1.03, 1.14)	0.004	1.07 (1.02, 1.13)	0.010	1.07 (1.02, 1.13)	0.012
Other	0.92 (0.88, 0.97)	<0.001	0.88 (0.84, 0.93)	<0.001	0.91 (0.87, 0.96)	<0.001	0.88 (0.83, 0.92)	<0.001
**Marital status**								
Unmarried	Reference		Reference		Reference		Reference	
Married	0.81 (0.78, 0.84)	<0.001	0.91 (0.88, 0.95)	<0.001	0.82 (0.79, 0.85)	<0.001	0.93 (0.89, 0.96)	<0.001
**Grade**								
Well/Moderate differentiation	Reference		Reference		Reference		Reference	
Poor differentiation/ Undifferentiation	1.14 (1.09, 1.20)	<0.001	1.24 (1.18, 1.30)	<0.001	1.15 (1.10, 1.21)	<0.001	1.24 (1.18, 1.30)	<0.001
**Tumour site**								
Cardia	Reference		Reference		Reference		Reference	
Body	1.13 (1.06, 1.21)	0.003	NA	NA	1.14 (1.06, 1.22)	<0.001	NA	NA
Lower	NA	NA	NA	NA	NA	NA	NA	NA
Overlapping lesion of stomach	1.22 (1.14, 1.30)	<0.001	1.18 (1.11, 1.26)	<0.001	1.22 (1.14, 1.30)	<0.001	1.18 (1.11, 1.26)	<0.001
Stomach NOS	1.37 (1.31, 1.44)	<0.001	1.18 (1.12, 1.24)	<0.001	1.36 (1.29, 1.43)	<0.001	1.17 (1.11, 1.24)	<0.001
**Chemotherapy**								
No	Reference		Reference		Reference		Reference	
Yes	0.37 (0.36, 0.39)	<0.001	0.36 (0.34, 0.37)	<0.001	0.38 (0.37, 0.39)	<0.001	0.36 (0.35, 0.38)	<0.001
**Surgery**								
No	Reference		Reference		Reference		Reference	<0.001
Yes	0.55 (0.52, 0.58)	<0.001	0.47 (0.44, 0.49)	<0.001	0.54 (0.52, 0.57)	<0.001	0.46 (0.44, 0.50)	<0.001
**Radiation therapy**								
No	Reference		Reference		Reference		Reference	
Yes	0.73 (0.69, 0.76)	<0.001	0.89 (0.85, 0.94)	<0.001	0.73 (0.70, 0.77)	<0.001	0.90 (0.85, 0.95)	<0.001

^a^only significant results presented (*P* < 0.05).

NOS, Not Otherwise Specified.

**Table 3:** Multivariate Cox analysis of gastrectomy for overall and cancer-specific survival stratified by age, chemotherapy and radiation^*^

Variable	Overall survival		Cancer-specific survival	
	HR (95% CI)	*P*	HR (95% CI)	*P*
**Age**				
<60	0.47 (0.43, 0.51)	<0.001	0.47 (0.43, 0.52)	<0.001
≥60	0.47 (0.44, 0.51)	<0.001	0.46 (0.43, 0.50)	<0.001
**Chemotherapy**				
No	0.56 (0.52, 0.60)	<0.001	0.55 (0.52, 0.60)	<0.001
Yes	0.45 (0.42, 0.49)	<0.001	0.45 (0.41, 0.49)	<0.001
**Radiation therapy**				
No	0.48 (0.45, 0.51)	<0.001	0.47 (0.45, 0.50)	<0.001
Radiation	0.40 (0.35, 0.46)	<0.001	0.41 (0.36, 0.47)	<0.001

^*^non-surgery group as reference.

Abbreviation: HR, hazard ratio; CI, confidential interval.

Adjusted for sex, race, age, grade, marital status, tumour site, radiation, chemotherapy.

### Surgery as a prognostic factor for survival after propensity score matched

**Figure 5:**
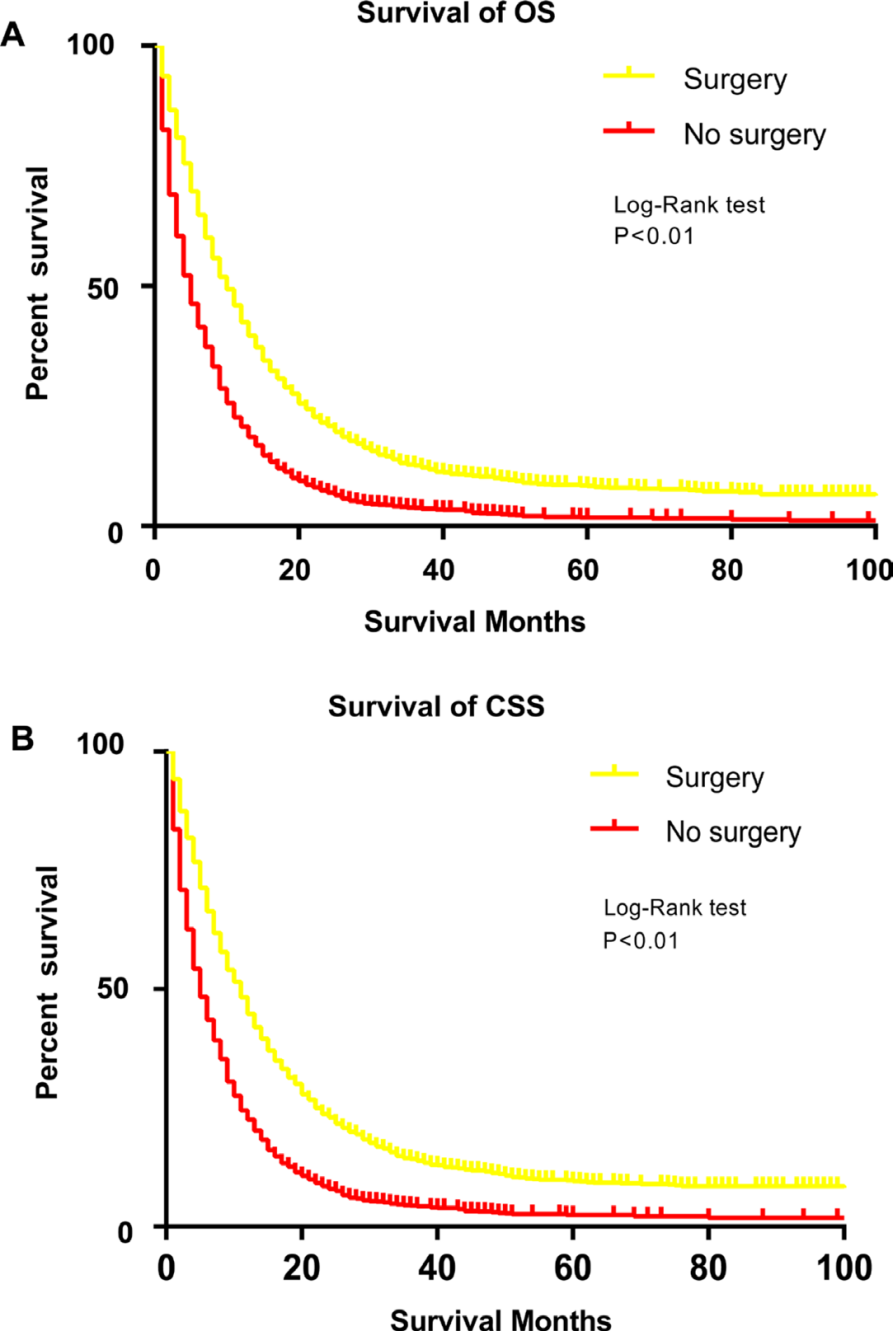
Survival analysis of patients with stage IV gastric cancer by Kaplan-Meier curves after propensity matching procedure (**A**) overall survival. (**B**) cancer-specific survival.

In order to reduce potential selection bias as inherent in previous studies, we performed a propensity score matching (PSM) for age, race, maritalstatus, tumour location, grade, chemotherapy and radiation. After 1:1 propensity score matching, 3962 patients were included in final analysis. By PSM, the baseline imbalance across groups had been reduced (Table 1). In the Cox analysis after propensity score matching, As shown in Figure 5A and 5B, patients who underwent gastrectomy enjoyed longer OS and CSS. In Cox analysis after PSM, gastrectomy persisted to be assosaiated with better OS (HR, 0.47, 95% CI, 0.43, 0.50) and CSS (HR, 0.47, 95% CI, 0.44, 0.50).

## DISCUSSION

In current study, we noted that the rate of gastrectomy in stage IV GC diminished over recent ten years, which was consistent with previous results [27]. We spectated that several causes might account for this trend. Firstly, palliative gastrectomy was not recommended for asymptomatic patients with stage IV according to European Society for Medical Oncology guideline [28]. Secondly, it might be due to development of other new systemic chemotherapies, which also improved the prognosis of these patients. Though the prognosis of patients with stage IV diseases was dismal for either surgery or non-surgery groups, our results revealed that gastrectomy significantly improved OS and CSS, even after adjusted for other variables. In addition, the survival benefits of gastrectomy persisted after propensity score matching, which further strengthen viability of our conclusion. Due to lacking of patients’ information about performance status, we were unable to adjust for this important confounding. Therefore, we compared the survival difference between surgery group and patients who were recommended but not performed surgery. We estimated that the performance status of surgery group and patients who were recommended to surgery might be similar. Gastrectomy still significantly decreased the overall mortality (HR, 0.56, 95% CI, 0.51–0.62) and cancer-specific mortality (HR, 0.55, 95% CI, 0.49–0.61). One strength of current study was large numbers of included patients and the results might mirror the real-world outcomes.

Despite the great achievements in the treatments of oncology, the outcome of advanced gastric cancer continues to be poor in the most areas [29]. During past decades, the value of gastrectomy in the setting of stage IV remained an ongoing debate. The practice of surgery in advanced gastric cancer was discouraged before 1980s because of relative high mortality and complication rates. Due to advancements in surgical techniques and managements of perioperative complications, surgery-related mortality was significantly reduced and the survival benefits were observed in recent studies. One meta-analysis [16] involving total 2,911 patients revealed that higher 1-year overall survival rate was observed in stage IV GC patients who underwent noncurative surgery. Besides, another meta-analysis [30] that included larger population also demonstrated that palliative gastrectomy could improve overall survival across patients with incurable gastric cancer.

Despite attractive better outcomes from observational studies, it must be of note that most evidence to date was based on retrospective studies, which introduce some bias invariably. Results from randomized clinical trials concerning this issue remained and inconstant. One RCT (GYMSSA trial) from the USA reported that the multimodal approach (including surgery) could improve survival among selected patients with gastric cancer [17]. In contrast, recently, newly completed randomized controlled trial included 330 patients (REGATTA trial) from Japan, South Korea, and Singapore revealed that gastrectomy plus chemotherapy for non-curable gastric cancer yielded no survival benefits in related to chemotherapy alone. Therefore, gastrectomy plus chemotherapy could not be justified with enough evidence in the clinical practiceC [8]. However, the conclusion from this RCT should be interpreted with caution since the trial included small patients. Early termination of the trial duo to futility also restricted the enough statistical power to demonstrate conclusions. In addition, the differences property of gastric cancer between Western and Asian populations might contribute to those disparities.

Our results also should be interpreted with caution and it was important to acknowledge that there were some the limitations cross the study. Firstly, we lacked information regarding performance status, comorbidities, detailed sites of distant metastasis, which might be critical for conclusion. Secondly, detailed information about chemotherapy regimens was not available in the SEER. Finally, it was impossible to avoid selection bias even if we performed PSM analysis. It was possible that patients who were healthier were likely to receive surgery, hence lead to better prognosis.

In conclusion, our study provided some evidence that patients with stage IV GC could benefit from gastrectomy. However, it remains too early to recommend surgery as a standard treatment for stage IV gastric cancer. Perspective trials need to examine the effect of gastrectomy for stage IV GC.

## MATERIALS AND METHODS

### Patient source and definition

We identified eligible patients from the Surveillance, Epidemiology, and End Results (SEER) database. We obtained detailed data by the SEER-stat software (SEER*Stat 8.3.1). Briefly, patients were included in the analysis as following: aged 18 years or older, diagnosis of gastric cancer with histologically confirmed stage IV, histology confirmed by using the International Classification of Diseases for Oncology (ICD-O-3, M-8140/3, M-8142/3 through M-8145/3, M-8210/3, M-8211/3, M-8255/3, M-8260/3 through M-8263/3, M-8310/3, M-8323/3, M-8480/3, M-8481/3, M-8490/3). We excluded patients from the analysis who lacked adequate information on surgery status or local resection and follow-up duration, patients with multiply primary cancers, case who were identified from autopsy or death certificate. Eligible population were classified according to whether they received primary cancer resection by site-specific surgery of primary site codes. Surgery group was divided into total (or near-total) gastrectomy (codes 40–42, 52, 60, 62, and 63) and non-total gastrectomy (any other code). The reason of not undergoing surgery was classified as “recommended but not performed”, “not recommended”, “not performed, patient died prior to surgery” according to SEER code about Reason no cancer-directed surgery. The study was exempt by the review board of the Sir Run Run Shaw Hospital because all data was public.

### Statistical analysis

Baseline demographic and clinical characterises were described by descriptive statistics and difference between the surgery and non-surgery groups were assessed by the chi square tests. To further reduce potential baseline bias in patient selection between two groups, we adopted 1:1 propensity score matching to re-examine the effect of resection. Confounders included in this propensity matching included age, gender, race, marital status, primary site, grade, radiation, chemotherapy. Afterwards, the matched patients were comparable with respect to the baseline characterises. Overall survival and cause-specific survival were evaluated by Kaplan–Meier curves and log-rank test. Univariable and multivariable Cox analysis were conducted to assess the prognostic effect of surgery in overall and cause-specific survival. A *P* value < 0.05 considered statistically significant and all statistical analyses were performed by SPSS version 20 (SPSS Inc, Chicago, USA), STATA version 13.0 (StataCorp, College Station, TX) and R version 3.4 (http://www.r-project.org).

## Abbreviations

SEER: Surveillance, Epidemiology and End Results
ICD-O-3: the third edition of the International Classification of Diseases for Oncology
PSM: Propensity score matching
HR: hazard ratio
CI: confidence interval
OS: overall survival
CSS: cancer-specific survival
SR: survival rate
GC: gastric cancer
